# Identifying and Improving Knowledge Deficits of Emergency Airway Management of Tracheotomy and Laryngectomy Patients: A Pilot Patient Safety Initiative

**DOI:** 10.1155/2010/638742

**Published:** 2010-05-26

**Authors:** Ivan H. El-Sayed, Susan Ryan, Hildy Schell, Rosanne Rappazini, Steven J. Wang

**Affiliations:** ^1^Department of Otolaryngology-Head and Neck Surgery, University of California, San Francisco, CA 94115, USA; ^2^Department of Anesthesia, University of California, San Francisco, CA 94115, USA; ^3^Department of Nursing, University of California, San Francisco, CA 94115, USA; ^4^Quality Improvement Department, University of California, San Francisco, CA 94115, USA

## Abstract

*Objectives*. To evaluate the knowledge base of hospital staff regarding emergent airway management of tracheotomy and laryngectomy patients, and the impact of the introduction of a bedside airway form. *Methods*. Cross-sectional surveys of physicians, nurses, and respiratory therapists at a tertiary care hospital prior to and 24 months after introduction of a bedside Emergency Airway Access (EAA) form. *Results*. Pre- and postintervention surveys revealed several knowledge deficits. Preintervention, 37% of medical internists and 19% overall did not know that laryngectomy patients cannot be orally ventilated, and 67% of internists could not identify the purpose of stay sutures in recently created tracheotomies. Postintervention, these numbers improved for all groups. Furthermore, 80% of respiratory therapists reported encountering the EAA form in an emergent situation and found it useful. *Conclusion*. A knowledge deficit is identified in caregivers expected to provide emergency management of patients with airway anatomy altered by subspecialty surgeons. Safety initiatives such as the EAA form may improve knowledge among providers.

## 1. Introduction

Treatment of acutely obstructed airways in a hospital setting is managed by primary emergent responders of the “Code Blue” team that may be comprised of a medical internist, anesthesiologist, surgeon, nurse, and respiratory therapist. Altered airway anatomy created by a subspecialist surgeon may not be immediately recognized nor understood, by other health care providers, potentially leading to poor outcomes. For instance, attempts of oral intubation in laryngectomy patients and occlusion of their tracheal stomas, or continued ventilation through a dislodged tracheotomy tube, can result in serious morbidity or mortality.

The issue of comprehension of airway anatomy amongst various providers was identified during multidisciplinary quality assurance reviews of Code Blue events at our institution after difficulties arose in the management of two patients with laryngectomies within a 12-month period. In one patient who suffered a cardiopulmonary arrest after total laryngectomy, oral intubation was the initial approach taken by the resuscitation team. During this review it became apparent that there was a lack of understanding of airway anatomy amongst many inpatient caregivers of these patients. Furthermore, communication between the airway experts (otolaryngologist-head and neck surgeons) and primary Code Blue responders (nurses, medical internists, anesthesiologists, general surgeons, and respiratory therapists) is limited by nonstandardized terminology, the rushed pace, and inherent confusion of these events. 

Because of the occurrence of these cases, a multidisciplinary team including faculty from the University of California, San Francisco Departments of Otolaryngology-Head and Neck Surgery; Anesthesia and Perioperative Care; and managers of the Departments of Nursing, Respiratory Care Services, and the UCSF Medical Center Performance Improvement staff initiated a quality improvement project designed to improve emergency airway management in patients who have previously undergone surgical alteration of their upper airway, typically tracheotomy or laryngectomy. 

The multidisciplinary team created a survey to assess the hospital staff's knowledge of emergency airway access in patients with surgically altered airways (tracheotomy or laryngectomy). After analysis of survey results and review of Code Blue records, a pilot program was introduced to improve knowledge of emergency airway management of these patients amongst inpatient caregivers. The program consisted of instructional lectures to relevant caregivers (anesthesiologists, general surgeons, internal medicine physicians, nurses, and respiratory therapists) and introduction of a bedside form to identify the altered airway. Since the difference between a tracheotomy and laryngectomy may not immediately be obvious to emergency responders, the bedside Emergency Airway Access form was developed to improve awareness and facilitate communication during emergency situations. 

Several groups have analyzed the type of instruction that is most effective in achieving house staff competence in emergency airway management [1], such as computerized patient stimulators [2] or independent practice combined with periodic feedback [3]. The utility of bedside posted forms, however, has not been analyzed. We describe our initial experience with a bedside airway form as part of a pilot patient safety initiative to identify and improve knowledge deficits of surgically altered airway management in a hospital setting.

## 2. Methods

### 2.1. Preintervention Survey

To assess the knowledge and understanding of surgically altered airways, an unblinded cross-sectional survey of UCSF Medical Center health care providers was conducted on a specific day in May 2004 and again in May 2006. During this day, physicians, nurses, and respiratory therapists in the perioperative areas, inpatient wards, and intensive care units of the UCSF Medical Center were invited to participate in a survey assessing knowledge of emergency airway access. The paper questionnaires contained written instructions to minimize communication between surveyors and participants, who generally completed the surveys on their own and returned them immediately. In some cases, surveyors returned later in the day to collect the questionnaires. All surveys were anonymous but had demographic questions regarding caregiver type, specialty, hospital site, and year of practice/training. The survey included 6 questions that tested understanding of the following points.

1. Laryngectomy patients cannot be ventilated orally.
2. Tracheotomy patients can be ventilated oronasally if the tracheotomy is cuffless or deflated.
3. It is not acceptable to continue to attempt ventilation of a displaced or plugged tracheotomy tube.
4. It is not acceptable to blindly reinsert a displaced tracheotomy tube before the stoma tract is fully matured.
5. The purpose of tracheal stay sutures is to allow for easier reinsertion of a displaced tracheotomy tube.

### 2.2. Intervention

**Figure 1:**
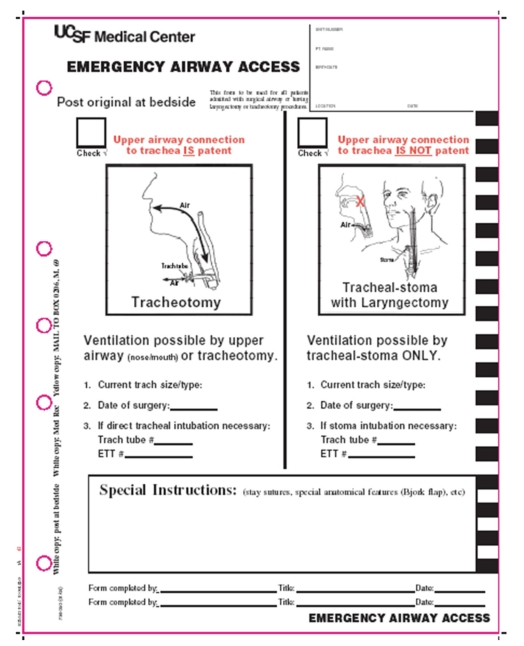
The UCSF Emergency Airway Access Form, posted at the bedside of all patients with surgically altered airways (reprinted with permission of UCSF and the Department of Otolaryngology-Head and Neck Surgery).

A new Emergency Airway Access (EAA) form for patients with surgically altered airways was created (Figure 1). This form included information on whether there was an available naso-oral airway in case the surgical airway in the neck was obstructed. Other basic information such as the presence of tracheal stay sutures or a Bjork flap was included along with the date, size, and type (cuffed/cuffless) of tracheotomy tube. This form was required to be posted at the patient bedside at all times during his/her stay in the hospital in order to facilitate daily and emergent care.

In addition to the bedside form, 10-minute instructional lectures were given at grand rounds of the UCSF Departments of Surgery, Medicine, Otolaryngology-Head and Neck Surgery, Anesthesiology, Nursing, and Respiratory Therapy. These lectures included a delineation of the airway form and covered all the points in the preintervention survey listed above. In-service training was given by clinical nurse instructors to nurses on all the wards and intensive care units that may care for tracheotomy and laryngectomy patients. Special training was provided to the Respiratory Therapy service by an otolaryngology attending physician including lectures on surgical airway anatomy and head and neck anatomy cadaver prosections. Respiratory therapists were trained to evaluate and recognize a patient having undergone laryngectomy procedures. A plan was introduced to identify patients with surgically altered airways by the respiratory therapy team at all points of hospital access (from the operating room, emergency room, and hospital transfers) and ensure that a form is posted at the head of the bed. Lastly, a UCSF medical center nursing newsletter article was dispersed announcing the new airway form, detailing its purpose and implementation.

### 2.3. Postintervention Survey

Twenty four months after the implementation of the airway form, another unblinded cross-sectional survey of the health care providers was performed, in a manner similar to the preintervention survey. This new survey included all the previous 6 airway related questions as well as additional questions regarding:

1. knowledge of the Airway Form's existence,
2. frequency of caregivers encountering emergent airway situations involving surgically altered airways,
3. utility of the form in an emergent airway situation,
4. receipt of training or education on emergent airway situations involving surgically altered airways.

### 2.4. Data Analysis

Percentages of correct answers were tallied for the two random cross sectional surveys and analyzed by Mantel-Haenszel Chi square test to assess the efficacy of airway forms and didactics to improve knowledge of management of surgically altered airways in the emergent setting. Differences across the two surveys were considered significant if the *P* value was less than .05. The data was also analyzed within the demographic subgroups of caregiver type (physician, nurse, respiratory therapist) and physician specialty (anesthesiology, internal medicine, surgery).

## 3. Results

**Figure 2:**
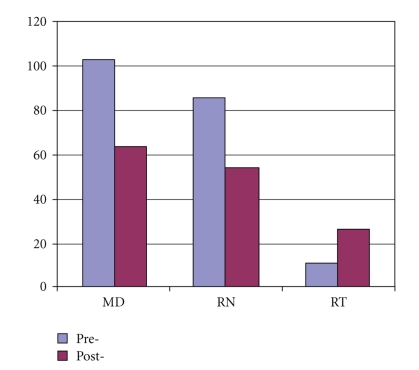
Caregiver type among survey participants.

**Figure 3:**
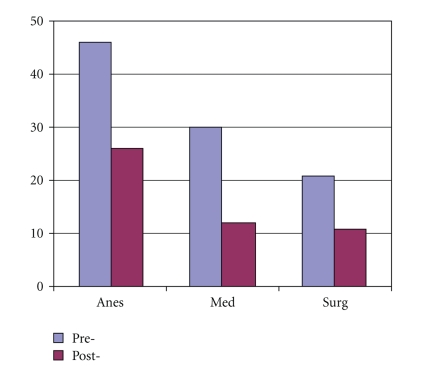
Physician specialty among survey participants.

A total of 200 physicians, nurses, and respiratory therapists took the first survey, while a fewer number of caregivers (144) took the postintervention survey (Figure 2). Physicians were comprised of attending physicians and residents from anesthesia, internal medicine, and general surgery. Among physician participants, more anesthesiologists were represented in the study than those from medicine or surgery (Figure 3). The second survey had less physicians and nurses participating, but more respiratory therapists.

**Figure 4:**
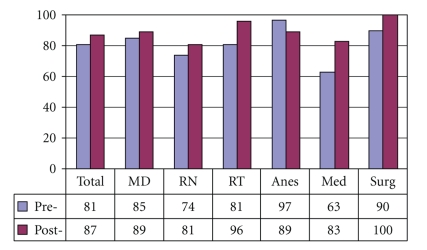
Laryngectomy patients have no nasal/oral airway (% Correct).

**Figure 5:**
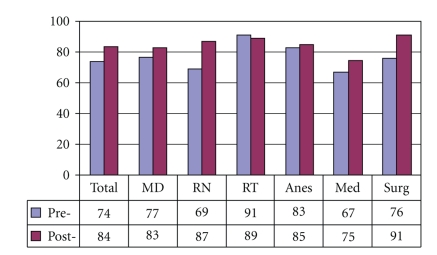
Tracheotomy patients can have an oral airway if tube is cuffless or cuff is deflated (% Correct).

**Figure 6:**
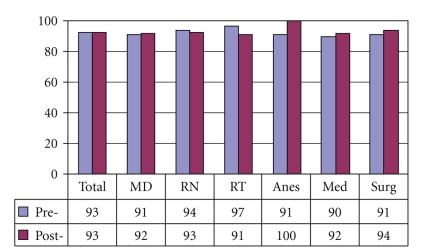
Do not blindly reinsert recently operated tracheotomy tubes (% Correct).

**Figure 7:**
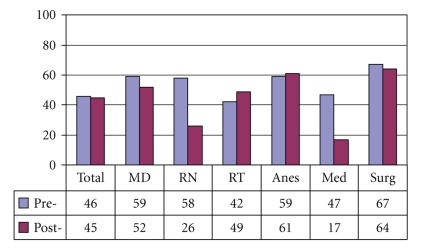
Do not continue ventilating an obstructed or displaced tracheotomy tube (% Correct).

**Figure 8:**
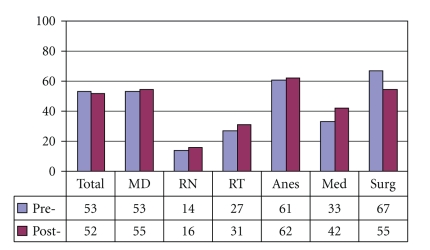
Stay sutures allow easier reinsertion of tracheotomy tube (% Correct).

Figure 4 demonstrates that preintervention, 37% of medical internists and 19% overall, did not understand that laryngectomy patients no longer have an oral or nasal airway. There was an overall improvement of laryngectomy airway anatomy knowledge in the intervening 24 months. There was also an overall improvement in the understanding that tracheotomy patients can be ventilated orally if the tracheotomy tube is cuffless or deflated (Figure 5). Figure 6 confirms that the majority of caregivers appreciate the danger of blind tube reinsertion in “fresh” tracheotomies. Figure 7 reveals that less than half of caregivers knew to discontinue the futile ventilation of a dislodged or plugged tracheotomy tube and to deflate the tracheotomy cuff for oral ventilation. In addition, nearly half of respondents did not know the purpose of “stay sutures” in a new tracheotomy wound in the event of accidental tube dislodgement (Figure 8). 

 The results of the pre- and postintervention surveys were compared and analyzed for statistically significant changes. No statistically significant differences (*P* > .05) could be appreciated using the Mantel-Haenszel Chi squared test.

**Figure 9:**
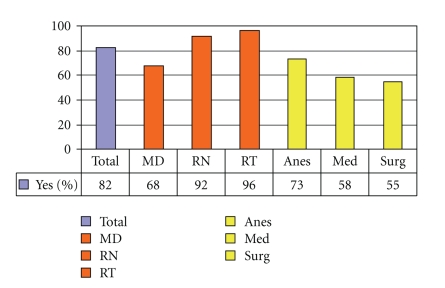
Aware of Emergency Airway Access Form.

**Figure 10:**
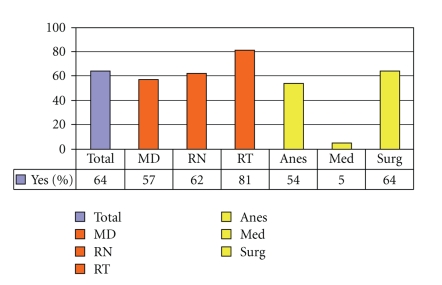
Have had ≥1 emergent situations with an altered airway in the last 12 months.

**Figure 11:**
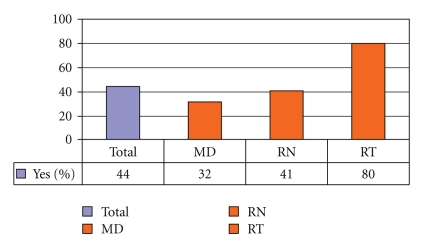
Found Emergency Airway Form helpful in the situation?

**Figure 12:**
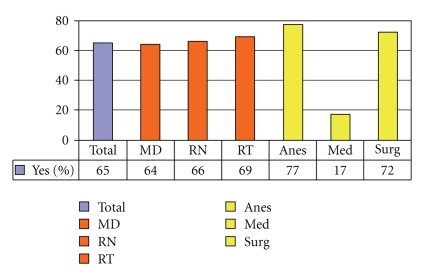
Received education/training on emergent airway situations in last 12 months?

The majority of hospital caregivers were aware of the EAA form twenty-four months after its introduction, but medical internists and general surgeons were least aware of the forms' existence (Figure 9). Figure 10 demonstrates that every group except for internal medicine physicians encounters substantial numbers of emergent situations involving patients with altered airways. Predictably, respiratory therapists encountered these events with the highest frequency. They were also the group that found the EAA form most helpful in these situations (Figure 11). A high proportion of all groups, except for the medical internists, reported recent education/training on emergent airway management (Figure 12).

## 4. Discussion

Patients who have undergone tracheotomy or laryngectomy have a risk of acute respiratory obstruction due to such common occurrences such as mucous plugging and tube displacement. After tracheotomy, the rate of serious complication is reported at 2.7% for tube obstruction and 1.5% for tube displacement [4]. A recent study reviewing 1130 tracheotomies found a death rate of 0.35%, which was most often caused by hemorrhage or tube displacement [5]. In a review of 183 laryngectomy patients, there was a 7% chance of airway complications, mostly thick mucous plugging associated with lack of humidification [6]. However, little data is reported on the exact nature and sequence of events that result in serious morbidity and mortality in this patient group. 

Airway emergencies are characterized by hypoxia or anoxia that can produce irreversible brain damage in a matter of minutes. Subspecialists, such as otolaryngologist-head and neck surgeons, routinely perform alterations to the upper airway that may not be immediately obvious to other medical and surgical providers who are often the primary responders of a typical code team. For instance, at the time of tracheotomy, a Bjork flap may be created or “stay sutures” may be placed in the upper and lower rings of the trachea to facilitate retraction of the rings for reinsertion of a dislodged tracheotomy tube. However, if primary responders do not know how to use “stay sutures,” these potential life-saving interventions are of little value. In addition, a laryngectomy tracheal stoma may be mistaken for a tracheotomy site. In this situation, oral ventilation or intubation may be attempted. Thus, it is important for medical, nursing, and respiratory therapy colleagues to comprehend key points of airway anatomy in these patients to avoid significant morbidity.

As a result of our hospital quality assurance process, we identified a potential knowledge deficit among typical primary responders in the code team. This study represents an attempt to accrue pilot data to characterize the baseline knowledge of caregivers at a tertiary care hospital and present our attempted solution to improve communication among providers.

During quality assurance review of events in prior Code Blue events involving laryngectomy patients at our institution, two important factors were identified that could impact patient outcomes: first, a lack of understanding of subspecialty alteration of the airway by other health care providers, and second, difficulty communicating this knowledge in a timely fashion due to the inherent limitations imposed in a Code Blue event. Our initial hospital staff survey identified specific knowledge gaps of airway anatomy, most notably among medical internists. Having identified a potential patient safety concern, our institution tasked a multidisciplinary committee to develop recommendations for improving the acute management of emergent airway situations in patients with altered airways. Loss of the airway can rapidly result in patient demise, and there is not always time for a knowledgeable airway specialist (i.e., head and neck surgeon) to respond. Thus, a pilot program was initiated to identify all surgically altered airways in the hospital and determine their airway status (i.e., tracheotomy, laryngectomy, or other high-risk airways such as patients who might be very difficult to orally intubate). These identified patients would have an Emergency Airway Access form posted at the bedside at all times (Figure 1). Since it was determined that a respiratory therapist is reliably present at every code, we focused particular attention to training this group of caregivers regarding the form, with the expectation that they could communicate any significant or unusual airway anatomy to the other caregivers at the code. 

Our data reveals that the majority of caregivers appreciate the danger of blind reinsertion in “fresh” tracheotomies (Figure 6). However, this knowledge does not always translate into clinical application. For instance, Figure 7 reveals that even though the majority of caregivers appreciate the danger of creating a false tract through blind reinsertion of the tracheotomy tube, less than half of medical internists knew to discontinue the futile ventilation and to deflate the tracheotomy cuff for oral ventilation. In addition, nearly half of respondents and 77% of medical internists did not know how to use “stay sutures” in a new tracheotomy wound in the event of accidental tube dislodgement (Figure 8). Given that medical internists are frequently in charge of Code Blue teams in these emergency settings, these findings are especially concerning.

Postintervention surveys showed overall improvement in knowledge regarding basic airway anatomy in patients after tracheotomy and laryngectomy. While the improvement in knowledge is gratifying, one could argue that close to 100% knowledge of airway anatomy should be expected among caregivers who may be called in an emergency to manage these patients. Regarding appropriate management of common emergency airway scenarios with laryngectomy and tracheotomy patients, the overall results did not improve after implementation of the bedside airway form. These findings suggest additional training regarding appropriate management of common emergency airway scenarios that occur with laryngectomy and tracheotomy patients may be needed in order to prevent future morbidity and mortality.

### 4.1. Study Limitations

This study presents pilot data assessing the knowledge base of caregivers involved in Code Blue settings and introduces the concept of the EAA form. The form was not meant to replace provider knowledge but was developed to help standardize terminology and improve communication efficiently in the rushed Code Blue setting. There are several limitations to our study. Unfortunately, at a teaching hospital, there is a significant amount of turnover of residents, nurses, and attending physicians. Thus, the data does not represent the same respondents pre- and postintervention. Statistical analysis in this setting is very weak due to the poorly controlled nature of the study, and we cannot conclude that the improvement in responses is due to the EAA form. However, the descriptive data does clearly identify a persistent knowledge gap among many providers in this setting. 

Despite the study's limitations, it nonetheless suggests the need for more education regarding appropriate emergent airway management of patients with altered airways as well as the need for more focused instruction regarding how the bedside airway form can be useful to assist critical clinical decision-making, especially by physicians who may be part of the code team. Improvements in tests of knowledge are only indirect measures of quality improvement. A true test of efficacy of the EAA would be to demonstrate a reduced mortality of airway events; however, given the rarity of these events, there is insufficient data to analyze at one institution.

## 5. Conclusion

Knowledge of the management of airway emergencies in patients with tracheotomies and laryngectomies among hospital staff is crucial to avoid unnecessary morbidity and mortality. Two successive surveys identified knowledge deficits among providers responsible for emergency airway management in Code Blue settings in these patients. Our pilot patient safety initiative program was designed with the purpose of improving communication in critical situations among providers. A key component of the program, the bedside EAA form, may also have improved understanding of basic airway anatomy of laryngectomy and tracheotomy patients. Our results suggest, however, that there is still need for more education regarding appropriate emergency airway management of patients with altered airways and for more training regarding how the EAA bedside form can be used to assist critical decisions in these situations.
